# Effect of vitamin D administration on sepsis score and C-reactive protein (CRP) levels in preterm infants with neonatal sepsis: a randomized controlled trial

**DOI:** 10.1186/s12887-026-06844-0

**Published:** 2026-05-11

**Authors:** Michelle Angelica Wijaya, Fiva Aprilia Kadi, Reni Ghrahani

**Affiliations:** Department of Child Health, Faculty of Medicine, Universitas Padjadjaran/Dr. Hasan Sadikin General Hospital, Bandung, West Java Indonesia

**Keywords:** Adjuvant therapy, Neonatal sepsis, Preterm infants, Vitamin D

## Abstract

**Background:**

Neonatal sepsis remains a leading cause of mortality in preterm infants. While Vitamin D possesses immunomodulatory properties, evidence for its adjuvant role in sepsis is limited. This study evaluated the efficacy of two Vitamin D doses (400 IU vs. 800 IU) on clinical sepsis scores and C-reactive protein (CRP) levels.

**Methods:**

An open-label, randomized controlled trial was conducted in Indonesia (August–December 2025) involving preterm infants (28–36 weeks) with sepsis. Patients were randomized into three groups for 7 days: (1) Controls (antibiotics only, nil per os [NPO]), (2) Vitamin D 400 IU/day, and (3) Vitamin D 800 IU/day. The open-label design was necessary due to the difference in feeding status (enteral vs. NPO). Outcomes included changes in the modified Töllner score, Sepsis Prediction Score (SPS), and serum CRP. Analysis was performed using ANCOVA and UNIANOVA, with laboratory analysts blinded to group allocation to strengthen the internal validity.

**Results:**

A total of 78 neonates were enrolled and randomized. After exclusions (*n* = 17), the final analysis included 61 neonates (Control: *n* = 23; 400 IU: *n* = 19; 800 IU: *n* = 19) with largely comparable baseline characteristics. Baseline Vitamin D deficiency was prevalent (73.7%). Both intervention groups showed significantly greater improvements in modified Töllner score and SPS compared to controls (*p* < 0.001), with no significant difference between the 400 IU and 800 IU doses (*p* = 1.000). Although CRP levels stabilized in the intervention groups and gained a significant increase in controls, adjusted inter-group differences at day 7 were not significant (*p* = 0.421). Mortality was higher in the control group (48.6%) compared to the intervention groups (*p* < 0.001); however, this is likely influenced by the greater baseline severity and cardiovascular instability inherent to their NPO status. Reported adverse events (Grade 1) did not require protocol cessation.

**Conclusion:**

Vitamin D administration (400 and 800 IU/day) significantly improves sepsis scores in preterm infants with comparable clinical effectiveness between doses, supporting its potential as an effective adjuvant therapy in neonatal sepsis.

**Trial registration:**

ClinicalTrials.gov NCT07245277. Registered 22 September 2025 - Retrospectively registered.

**Supplementary Information:**

The online version contains supplementary material available at 10.1186/s12887-026-06844-0.

## Background

Neonatal sepsis is a primary cause of global neonatal morbidity and mortality (1.3 million cases, 203,000 deaths annually), with a higher incidence in developing countries like Indonesia [1, 2]. Preterm infants are particularly vulnerable due to an immature immune system (antibody, complement, and neutrophil deficiencies), leading to higher mortality rates (up to 20%) and long-term morbidity [3, 4]. Diagnosis is often difficult due to non-specific symptoms, necessitating biomarkers like C-reactive protein (CRP) and scoring systems (modified Töllner score, Sepsis Prediction Score [SPS]) [5, 6]. Various adjuvant therapies have been explored, but clinical evidence remains limited [7–9]. 

Vitamin D is known for its crucial immunomodulatory role, regulating both innate (via antimicrobial peptides) and adaptive immunity [10]. Despite its potential as an affordable and safe adjuvant therapy, the prevalence of vitamin D deficiency in neonates is ironically high globally (61–90%) [11, 12]. Studies on the effectiveness of vitamin D as an adjuvant for neonatal sepsis, especially in Indonesia, are scarce. Furthermore, no specific dose recommendations exist for neonatal sepsis. Therefore, this study aims to evaluate the effectiveness and compare the dosing (400 IU vs. 800 IU) of vitamin D administration on clinical outcomes (sepsis scores) and CRP levels in preterm infants with sepsis.

## Materials and methods

### Study aim

This study aims to determine differences in sepsis scores (modified Töllner score, SPS) and CRP levels between preterm infants with sepsis receiving adjuvant vitamin D therapy at 400 IU/day or 800 IU/day and a control group receiving only antibiotics.

### Study design

This study was an analytical experimental study utilizing a randomized controlled trial (RCT) design. The reporting of this trial adheres to the CONSORT (Consolidated Standards of Reporting Trials) guidelines. The study was conducted at the Neonatal Intensive Care Unit (NICU) and Neonatal High Care Unit (NHCU) of Dr. Hasan Sadikin General Hospital and Bandung Kiwari Regional Hospital, Indonesia. Inclusion criteria were preterm infants (gestational age 28–36 weeks) diagnosed with sepsis (positive blood culture) or probable sepsis (modified Töllner score ≥ 5 or SPS ≥ 3) whose parents provided informed consent. Exclusion criteria included major congenital malformations (anencephaly, encephalocele, holoprosencephaly, hydrocephalus, meningomyelocele, spina bifida, omphalocele, gastroschisis, or congenital heart disease) or significant changes in feeding status (e.g., nil per os [NPO] to enteral or vice versa) during the 7-day monitoring period. Subjects were allocated into three groups.

- Group 1 (Control): Antibiotics only (required NPO).
- Group 2 (Intervention): Antibiotics plus 400 IU/day of Vitamin D3 drop 400 IU/0.5 mL for 7 days (receiving enteral nutrition).
- Group 3 (Intervention): Antibiotics plus 800 IU/day of Vitamin D3 drop 400 IU/0.5 mL for 7 days (receiving enteral nutrition).

The study received ethical approval, and informed consent was obtained from all parents or guardians.

The allocation of the control group was guided by ethical considerations, because the routine vitamin D administration is recommended for all neonates, it was deemed unethical to withhold oral vitamin D drops from a control group of neonates who were tolerating enteral nutrition. Consequently, the control group exclusively comprised neonates who were strictly NPO due to medical necessity, as they were unable to receive oral administration.

Safety was monitored daily by the clinical research team. Predefined stopping rules included the occurrence of confirmed severe hypercalcemia (serum calcium > 11.5 mg/dL) on repeated testing, any Grade 3 or higher adverse event according to Common Terminology Criteria for Adverse Events (CTCAE), or clinical deterioration where the attending neonatologist deemed discontinuation necessary. No participants met the criteria for study withdrawal due to adverse events during the trial period.

Randomization was performed using a computer-generated random number sequence. Allocation concealment was not implemented; the primary investigator generated the randomization sequence and performed the subsequent enrollment and assignment of participants to their respective groups. Although the clinical administration of the intervention was open-label due to the nature of nutritional status (NPO vs. enteral), the outcome assessors, specifically the laboratory analysts measuring CRP levels, were blinded to group assignments to minimize detection bias. Patients and the public were not involved in the design, conduct, or reporting of this research. There were no changes to the methods or trial outcomes after the trial commenced. The trial ended when the target sample size was reached. This trial was retrospectively registered on ClinicalTrials.gov under the identifier NCT07245277, with an initial release date of 22 September 2025 and a public release date of 17 November 2025.

### Sample size determination

The sample size was calculated to detect a significant difference in CRP levels between groups using the formula for comparing independent-group means (unpaired numerical design). Based on a previous reference study by Hagag et al. [13], we assumed a common standard deviation (SD) of 1.53 mg/dL and a minimum clinically meaningful difference in CRP levels of 1.26 mg/dL. The calculation used a one-sided hypothesis test with a 95% confidence level (α = 0.05, Zα = 1.64) and 80% power (β = 0.20, Zβ = 0.84). Based on these assumptions, the minimum required sample size was determined to be 19 neonates per group, yielding a total of 57 neonates across the three study groups.

### Data collection

Subjects were enrolled using consecutive sampling, and primary data alongside subject characteristics were obtained from patient medical records. Venous blood samples (2 mL) were collected at baseline (day 0) and post-intervention (day 7). Laboratory analyses included hematology and CRP. Serum CRP levels were measured using quantitative fluorescence immunochromatography assay (Zybio Inc., Chongqing, China) on a fluorescence immunoassay analyzer. In this study, repeated CRP testing was performed on day 7, although serial monitoring on day 3 is considered ideal. This constraint was due to limitations in blood sampling and laboratory resources at our hospital, which restrict repeated testing to intervals of at least 7 days for neonates who do not exhibit clinical deterioration.

Meanwhile, serum 25(OH)D levels were assessed only at day 0 and were measured using a Competitive Enzyme-Linked Immunosorbent Assay (ELISA), as repeated testing was precluded by resource and financial limitations. Clinical sepsis scores (modified Töllner, SPS) were also recorded at day 0 and day 7. While the primary intervention and clinical data collection occurred over this 7-day period, neonates were followed up continuously, and clinical outcomes including mortality were recorded until their discharge from the hospital. There were no changes to the methods or trial outcomes after the trial commenced.

### Statistical analyses

Data were analyzed using SPSS version 27.0. Descriptive statistics were used for baseline characteristics, and normality was assessed using the Shapiro-Wilk test. The primary analysis for comparing group outcomes (CRP, sepsis scores) was Analysis of Covariance (ANCOVA) and Univariate Analysis of Variance (UNIANOVA). In this model, the day 7 value was the dependent variable, the treatment group was the independent variable, and the baseline (day 0) value was included as a covariate. Prior to performing ANCOVA, the required assumptions were verified: linearity of the relationship between the covariate and the dependent variable was assessed via scatterplots, homogeneity of regression slopes was confirmed by checking the interaction between the treatment groups and the covariate, and homogeneity of variances was evaluated using Levene’s test. Additionally, the normality of model residuals was verified. Results are reported as adjusted mean differences with 95% confidence intervals (CIs) and effect sizes (partial eta squared, *ηp*²). A p-value of ≤ 0.05 was considered statistically significant.

## Results

### Baseline characteristics

A total of 78 neonates were enrolled, of whom 61 were included in the final analysis. Seventeen patients were excluded (nine deaths before 7-day monitoring and eight due to changes in feeding status). Baseline characteristics were generally comparable among the three groups (control, 400 IU, and 800 IU). There were no statistically significant differences found in gender (*p* = 0.604), chronological age (*p* = 0.074), gestational age (*p* = 0.834), mean birth weight (*p* = 0.649), or the distribution of respiratory support (*p* = 0.965). Baseline vitamin D deficiency was highly prevalent (73.7% of all patients), reaching 91.3% in the NPO control group. While baseline vitamin D levels trended lower in the control group (median 9.36 ng/mL) compared to the 400 IU (16.35 ng/mL) and 800 IU (20.50 ng/mL) groups, this difference did not reach statistical significance (*p* = 0.060). Most additional clinical characteristics were well-balanced across the three groups (*p* > 0.05), including blood culture positivity (36.8%–47.8%), microbiological profiles (predominantly *Acinetobacter baumanii* and *Klebsiella pneumoniae*), antibiotic usage, and the prevalence of comorbidities such as respiratory distress syndrome (RDS), necrotizing enterocolitis (NEC), and intraventricular hemorrhage (IVH). Surfactant administration also showed no significant differences. However, a significant difference was observed in the requirement for inotropic support (*p* = 0.017), with a higher prevalence in the control group (69.6%) compared to the Vitamin D 400 IU (42.1%) and 800 IU (26.3%) groups (Table 1).

**Table 1:** Baseline characteristics of analyzed groups

Variable	Control (*n* = 23)	Vitamin D 400 IU (*n* = 19)	Vitamin D 800 IU (*n* = 19)	*p* value
Gender, n (%)				0.604ᶜ
Male	12 (52.2%)	12 (63.2%)	9 (47.4%)	
Female	11 (47.8%)	7 (36.8%)	10 (52.6%)	
Age (days), Median (IQR)	4 (2–11)	10 (5–20)	6 (4–13)	0.074ᵇ
Gestational age (weeks), Median (IQR)	33 (29–34)	32 (29–33)	32 (32–33)	0.834ᵇ
Gestational age category, n (%)				0.417^b^
32–<37 weeks	9 (39.1%)	7 (36.8%)	4 (21.1%)	
28–<32 weeks	14 (60.9%)	12 (63.2%)	15 (78.9%)	
Birth weight (gram), Mean ± SD	1579.5 ± 568.3	1536.7 ± 386.4	1674.3 ± 397.0	0.649ᵃ
Birth weight category, n (%)				0.417^b^
< 1000 gram	6 (26.1%)	2 (10.5%)	1 (5.3%)	
1000–<1500 gram	4 (17.4%)	7 (36.8%)	3 (15.8%)	
1500–<2500 gram	11 (47.8%)	10 (52.6%)	15 (78.9%)	
≥ 2500 gram	2 (8.7%)	0 (0.0%)	0 (0.0%)	
Respiratory support, n (%)				0.965ᶜ
Low Flow	1 (4.3%)	1 (5.3%)	1 (5.3%)	
CPAP/NIV	5 (21.7%)	5 (26.3%)	6 (31.6%)	
Mechanical ventilator	17 (73.9%)	13 (68.4%)	12 (63.2%)	
Vitamin D (ng/mL), Median (IQR)	9.36 (5.27–13.60)	16.35 (6.64–28.30)	20.50 (8.10–24.99)	0.060ᶜ
Vitamin D status classification, n (%)				0.038ᶜ*
Sufficient	3 (13.0%)	4 (21.1%)	4 (21.1%)	
Insufficient	0 (0.0%)	1 (5.3%)	5 (26.3%)	
Deficient	20 (87.0%)	14 (73.6%)	10 (52.6%)	
Blood culture, n (%)				0.772ᶜ
Positive	11 (47.8%)	8 (42.1%)	7 (36.8%)	
Negative	12 (52.2%)	11 (57.9%)	12 (63.2%)	
Blood culture result (organism), n (%)				0.343ᶜ
* Acinetobacter baumanii*	7 (30.4%)	1 (5.3%)	4 (21.1%)	
* Klebsiella pneumoniae*	2 (8.7%)	5 (26.3%)	1 (5.3%)	
* Stenotrophomonas maltiphilia*	0 (0.0%)	1 (5.3%)	1 (5.3%)	
* Candida albicans*	1 (4.3%)	0 (0.0%)	1 (5.3%)	
MRCoNS	1 (4.3%)	0 (0.0%)	0 (0.0%)	
* Ralstonia pickettii*	0 (0.0%)	1 (5.3%)	0 (0.0%)	
Negative	12 (52.2%)	11 (57.9%)	12 (63.2%)	
Antibiotic class use, n (%)				
Cephalosporins and Penicillins	14 (60.9%)	12 (63.2%)	13 (68.4%)	0.876ᶜ
Carbapenems	7 (30.4%)	5 (26.3%)	5 (26.3%)	0.941ᶜ
Aminoglycosides	17 (73.9%)	10 (52.6%)	11 (57.9%)	0.327ᶜ
Fluoroquinolones	2 (8.7%)	2 (10.5%)	2 (10.5%)	0.973ᶜ
Macrolides	1 (4.3%)	2 (10.5%)	0 (0.0%)	0.320ᶜ
Glycopeptides	0 (0.0%)	1 (5.3%)	1 (5.3%)	0.535ᶜ
Antifungal agents	14 (60.9%)	11 (57.9%)	11 (57.9%)	0.974ᶜ
Inotropes, n (%)				0.017ᶜ*
Yes	16 (69.6%)	8 (42.1%)	5 (26.3%)	
No	7 (30.4%)	11 (57.9%)	14 (73.7%)	
Comorbidity pattern, n (%)				0.205ᶜ
IVH	0 (0.0%)	0 (0.0%)	1 (5.3%)	
RDS	11 (47.8%)	8 (42.1%)	13 (68.4%)	
NEC	4 (17.4%)	1 (5.3%)	0 (0.0%)	
IVH + RDS	0 (0.0%)	1 (5.3%)	2 (10.5%)	
RDS + NEC	5 (21.7%)	5 (26.3%)	1 (5.3%)	
IVH + RDS + NEC	1 (4.3%)	0 (0.0%)	0 (0.0%)	
None	2 (8.7%)	4 (21.1%)	2 (10.5%)	
Surfactant, n (%)				0.114ᶜ
Yes	7 (30.4%)	5 (26.3%)	1 (5.3%)	
No	16 (69.6%)	14 (73.7%)	18 (94.7%)	

Numerical data *p*-values were calculated using

^a^ Independent t-test and

^b^ Mann–Whitney test

Categorical data *p*-values were calculated using

^c^ Chi-square test

*Significant *p*-value <0.05

### Comparison of sepsis scores and CRP levels between therapy groups

#### Overall analysis

**Fig. 1:**
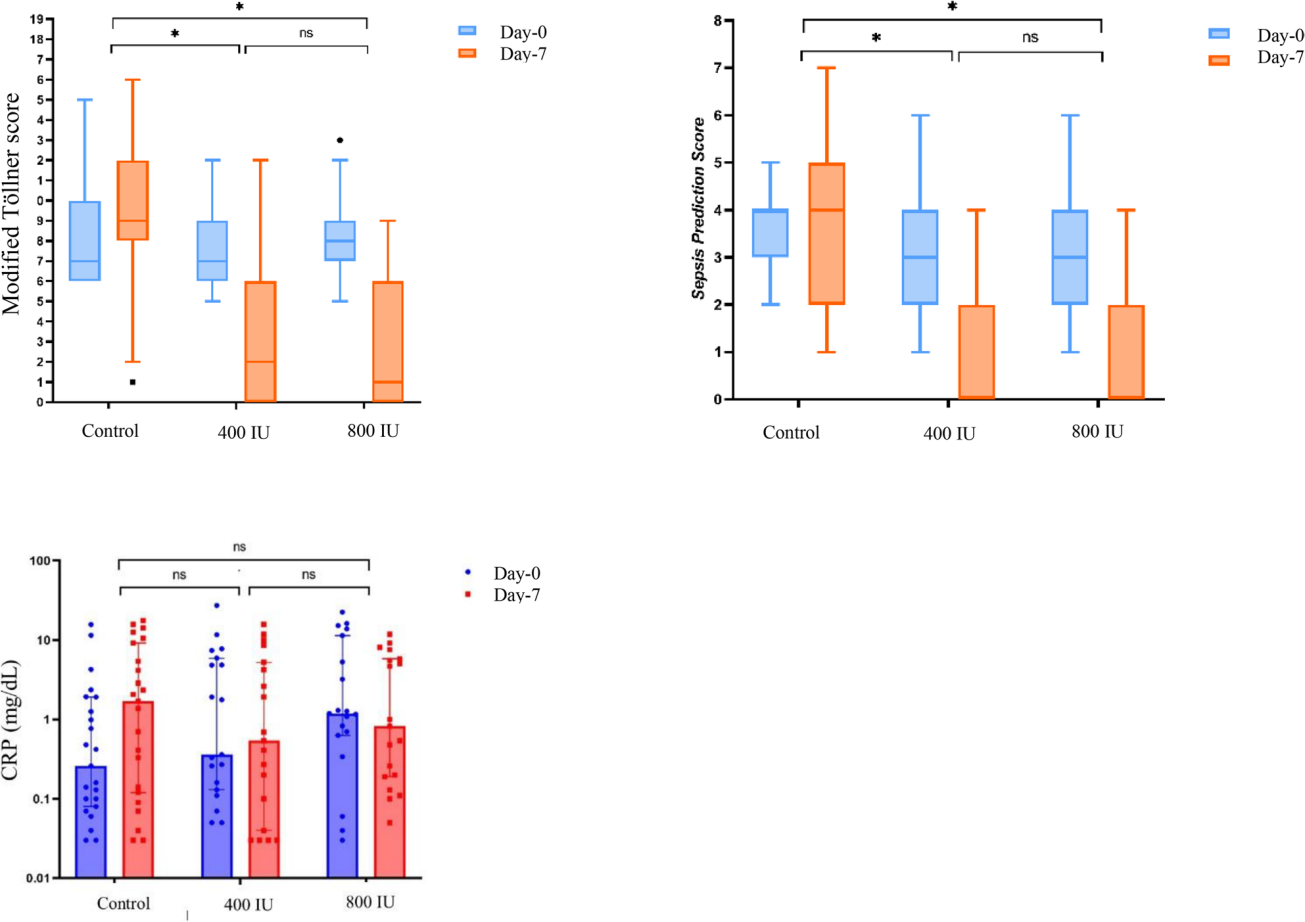
Comparison of Sepsis Scores and CRP Levels Between Therapy Groups. Data are presented as box plots indicating the median (horizontal line) and interquartile range (box). The whiskers represent the minimum and maximum values. The notation “ns” indicates a non-significant difference (*p* ≥ 0.05), while the asterisk (*) denotes a statistically significant difference (*p* < 0.05) between groups

The dynamics of sepsis scores and CRP levels from baseline (day 0) to day 7 showed distinct patterns among the groups (Table 2). For the modified Töllner score, the control group demonstrated a median increase from 7 (IQR 6–10) to 9 (IQR 8–12), with a median change (Δ) of + 1 (IQR 0–4, p = 0.130). In contrast, significant reductions were observed in the vitamin D groups; the 400 IU group decreased from a median of 7 to 2 (Δ − 5, p = 0.001) and the 800 IU group from 8 to 1 (Δ − 6, p < 0.001) (Fig. 1). Inter-group comparisons at day 7 and for the overall score change (Δ) were highly significant (both p < 0.001).

**Table 2:** Comparison of sepsis scores and CRP levels between therapy groups

Gestatio-nal age	Variable Outcomes	Intervention	Day–0 Median (IQR)	Day–7 Median (IQR)	Δ Day–0 to Day–7 Median (IQR)	*p*-value (pre–post)
Overall	Modified Töllner score	Control (*n* = 23)	7 (6–10)	9 (8–12)	1 (0–4)	0.130
		Vitamin D 400 IU (*n* = 19)	7 (6–9)	2 (0–6)	−5 (− 7–−1)	0.001*
		Vitamin D 800 IU (*n* = 19)	8 (7–9)	1 (0–6)	−6 (− 8–−2)	< 0.001*
		p-value (between groups)	0.275	< 0.001*	< 0.001*	
	Sepsis Prediction Score	Control (*n* = 23)	4 (3–4)	4 (2–5)	0 (− 1–1)	0.903
		Vitamin D 400 IU (*n* = 19)	3 (2–4)	0 (0–2)	−2 (− 3–−1)	0.001*
		Vitamin D 800 IU (*n* = 19)	3 (2–4)	0 (0–2)	−2 (− 3–−1)	< 0.001*
		p-value (between groups)	0.016*	< 0.001*	< 0.001*	
	CRP (mg/dL)	Control (*n* = 23)	0.26 (0.08–1.92)	1.71 (0.12–9.19)	0.25 (− 0.06–4.30)	0.039*
		Vitamin D 400 IU (*n* = 19)	0.36 (0.13–5.93)	0.54 (0.04–5.25)	−0.06 (− 4.16–1.82)	0.687
		Vitamin D 800 IU (*n* = 19)	1.19 (0.63–11.41)	0.82 (0.19–5.82)	−0.93 (− 7.99–0.42)	0.136
		p-value (between groups)	0.125	0.671	0.030*	
32–<37 weeks	Modified Töllner score	Control (*n* = 9)	7 (7–11)	10 (9–12)	2 (0.5–3)	0.055
		Vitamin D 400 IU (*n* = 7)	7 (5–10)	5 (0–9)	−5 (− 7–0)	0.058
		Vitamin D 800 IU (*n* = 4)	9.5 (8.25–12.25)	2.5 (1.25–4.5)	−6 (− 10.75–−5)	0.066
		p-value (between groups)	0.384	0.005*	0.002*	
	Sepsis Prediction Score	Control (*n* = 9)	4 (3–4.5)	4 (4–5.5)	1 (0–1.5)	0.038*
		Vitamin D 400 IU (*n* = 7)	3 (1–4)	0 (0–2)	−2 (− 4–−1)	0.017*
		Vitamin D 800 IU (*n* = 4)	3 (2.25–4.5)	0.5 (0–1.75)	−2 (− 4.5–−1)	0.066
		p-value (between groups)	0.475	0.001*	0.001*	
	CRP (mg/dL)	Control (*n* = 9)	0.14 (0.07–2.64)	2.86 (0.86–10.85)	2.72 (0.31–4.61)	0.008*
		Vitamin D 400 IU (*n* = 7)	1.77 (0.16–7.40)	0.20 (0.03–8.58)	−0.06 (− 1.36–1.18)	0.612
		Vitamin D 800 IU (*n* = 4)	3.25 (0.33–18.22)	2.85 (0.45–8.11)	−2.62 (− 11.04–3.26)	0.465
		p-value (between groups)	0.372	0.297	0.051	
28–<32 weeks	Modified Töllner score	Control (*n* = 14)	7 (6–7.75)	8 (5.5–12.25)	0.5 (− 3–4.25)	0.476
		Vitamin D 400 IU (*n* = 12)	7.5 (6.25–8)	1.5 (0.25–4.5)	−5.5 (− 7–−2.5)	0.005*
		Vitamin D 800 IU (*n* = 15)	8 (7–9)	1 (0–7)	−6 (− 8–−2)	0.001*
		p-value (between groups)	0.274	0.001*	< 0.001*	
	Sepsis Prediction Score	Control (*n* = 14)	4 (3–4)	3 (2–5)	−0.5 (− 2–1)	0.332
		Vitamin D 400 IU (*n* = 12)	3 (2–3)	0.5 (0–1.75)	−2 (− 3–−1)	0.009*
		Vitamin D 800 IU (*n* = 15)	3 (2–4)	0 (0–2)	−2 (− 3–−1)	0.001*
		p-value (between groups)	0.031*	< 0.001*	0.016*	
	CRP (mg/dL)	Control (*n* = 14)	0.45 (0.10–1.92)	0.56 (0.06–6.70)	−0.04 (− 0.41–2.44)	0.861
		Vitamin D 400 IU (*n* = 12)	0.32 (0.12–4.86)	1.31 (0.10–4.99)	−0.19 (− 4.48–4.36)	0.754
		Vitamin D 800 IU (*n* = 15)	1.16 (0.63–11.41)	0.54 (0.13–5.82)	−0.50 (− 7.99–0.42)	0.211
		p-value (between groups)	0.356	0.913	0.337	

Within-group comparisons (Day–0 vs. Day–7) were tested using the Wilcoxon signed-rank test. Between-group comparisons at each timepoint (Day–0, Day–7, and Δ) were tested using the Kruskal–Wallis test. The Δ (Delta) values represent the absolute change in sepsis scores and serum CRP levels, calculated by subtracting the post-intervention measurement (Day 7) from the baseline measurement (Day 0). Negative values indicate a reduction in CRP. Data are presented as mean ± standard deviation. *Significant p-value < 0.05*

Regarding the SPS, the control group median remained unchanged at 4 (p = 0.903), whereas both vitamin D groups showed significant reductions to a median of 0 by day 7 (both p ≤ 0.001). Although baseline SPS scores differed significantly between groups (p = 0.016), the magnitude of the decrease on day 7 was markedly greater in the intervention groups (inter-group Δ p < 0.001).

For CRP levels, the control group showed a significant increase from a median of 0.26 mg/dL to 1.71 mg/dL (p = 0.039). The vitamin D 400 IU group showed no significant change (p = 0.687), while the 800 IU group showed a downward trend from 1.19 mg/dL to 0.82 mg/dL, which did not reach statistical significance (p = 0.136). However, the overall change in CRP (Δ) differed significantly between therapy groups (p = 0.030).

#### Stratified analysis by gestational age

In the 32–<37 weeks subgroup, the control group showed a worsening trend in the Modified Töllner score (Δ + 2, p = 0.055), while the 400 IU and 800 IU groups showed reductions (Δ − 5 and − 6, respectively), though these intra-group changes did not reach statistical significance (p = 0.058 and p = 0.066). Nevertheless, inter-group differences remained significant at day 7 (p = 0.005) and for Δ (p = 0.002). The SPS in this subgroup worsened significantly in the control group (p = 0.038) but improved in the 400 IU group (p = 0.017). C-reactive protein levels in the control group increased significantly from 0.14 mg/dL to 2.86 mg/dL (p = 0.008), whereas no significant changes were observed in the vitamin D groups. The inter-group difference for ΔCRP in this subgroup approached, but did not reach significance (p = 0.051).

In the 28–<32 weeks subgroup, the reduction in sepsis scores was more consistent. The modified Töllner score decreased significantly in both the 400 IU (p = 0.005) and 800 IU (p = 0.001) groups, with inter-group differences remaining highly significant (p < 0.001). Similarly, both intervention groups showed significant reductions in SPS scores (p = 0.009 and p = 0.001), while the control group showed no significant change. No significant differences in CRP changes were observed across groups in this gestational subgroup (p > 0.05).

Overall, the administration of vitamin D at doses of 400 IU/day and 800 IU/day was associated with significant reductions in clinical sepsis scores compared to the control group across all analyses. While the inflammatory marker CRP demonstrated a significant increase in the control group, vitamin D administration appeared to stabilize or reduce these levels, particularly in the overall analysis and the 32–<37 weeks gestational subgroup.

### Adjusted comparison of sepsis scores and CRP levels

Based on the analysis of day-7 outcomes adjusted for baseline values (ANCOVA) and the analysis of change (Δ) without baseline adjustment (UNIANOVA), vitamin D administration was associated with significant clinical improvement in sepsis scores compared to the control group. However, no statistically significant effect was observed for CRP levels (Table 3).

**Table 3:** ANCOVA-adjusted comparison of Day-7 sepsis scores and CRP between therapy groups (Day-7 adjusted for baseline; Δ unadjusted)

Outcome	Group (*n*)	Adjusted mean / geometric mean (95% CI)	Comparison	Adjusted effect (95% CI)	*p*-valueᵃ	Overall group effect
Modified Töllner score (D-7)	Control (23)	9.35 (7.94–10.75)	Reference	—	—	F(2,57) = 24.394, *p* < 0.001; ηp²=0.461
	Vitamin D 400 IU (19)	3.65 (2.10–5.20)	Control – Vit D 400	5.70 (3.12–8.27)	< 0.001	
	Vitamin D 800 IU (19)	2.72 (1.17–4.27)	Control – Vitamin D 800	6.63 (4.05–9.20)	< 0.001	
			Vitamin D 800 – Vitamin D 400	0.93 (− 1.78–3.64)	1.000	
Δ Modified Töllner score (D-7 – D-0)	Control (23)	1.30 (− 0.13–2.74)	Reference	—	—	F(2,58) = 23.964, *p* < 0.001; ηp²=0.452
	Vitamin D 400 IU (19)	−4.32 (− 5.89–−2.74)	Control – Vitamin D 400	5.62 (3.00–8.24)	< 0.001	
	Vitamin D 800 IU (19)	−5.47 (− 7.05–−3.90)	Control – Vitamin D 800	6.78 (4.16–9.40)	< 0.001	
			Vitamin D 800 – Vitamin D 400	1.16 (− 1.58–3.90)	0.907	
Sepsis Prediction Score (D-7)	Control (23)	3.62 (3.07–4.18)	Reference	—	—	F(2,57) = 25.738, *p* < 0.001; ηp²=0.475
	Vitamin D 400 IU (19)	1.01 (0.41–1.62)	Control – Vitamin D 400	2.61 (1.57–3.65)	< 0.001	
	Vitamin D 800 IU (19)	1.08 (0.48–1.67)	Control – Vitamin D 800	2.55 (1.53–3.56)	< 0.001	
			Vitamin D 800 – Vitamin D 400	0.07 (− 0.97–1.10)	1.000	
Δ Sepsis Prediction Score (D-7 – D-0)	Control (23)	0.09 (− 0.51–0.68)	Reference	—	—	F(2,58) = 16.447, *p* < 0.001; ηp²=0.362
	Vitamin D 400 IU (19)	−2.05 (− 2.71–−1.40)	Control – Vitamin D 400	2.14 (1.05–3.23)	< 0.001	
	Vitamin D 800 IU (19)	−2.11 (− 2.76–−1.45)	Control – Vitamin D 800	2.19 (1.10–3.29)	< 0.001	
			Vitamin D 800 – Vitamin D 400	0.05 (− 1.09–1.20)	1.000	
CRP (mg/dL) (D-7)ᵇ	Control (23)	1.29 (0.54–3.05)	Reference	—	—	F(2,57) = 0.877, *p* = 0.421; ηp²=0.030
	Vitamin D 400 IU (19)	0.56 (0.22–1.42)	Vitamin D 400 vs. Control	GMR 0.43 (0.09–2.08)	0.584	
	Vitamin D 800 IU (19)	0.78 (0.30–2.00)	Vitamin D 800 vs. Control	GMR 0.60 (0.12–2.99)	1.000	
			Vit D 800 vs. Vitamin D 400	GMR 1.39 (0.27–7.03)	1.000	
Δ CRP (mg/dL) (D-7 – D-0)	Control (23)	2.56 (− 0.15–5.27)	Reference	—	—	F(2,58) = 2.618, *p* = 0.082; ηp²=0.083
	Vitamin D 400 IU (19)	−0.67 (− 3.65–2.31)	Control – Vitamin D 400	3.24 (− 1.72–8.19)	0.339	
	Vitamin D 800 IU (19)	−1.82 (− 4.80–1.16)	Control – Vitamin D 800	4.38 (− 0.58–9.34)	0.100	
			Vit D 800 – Vitamin D 400	1.15 (− 4.04–6.34)	1.000	

Model diagnostics (brief)

• Levene’s test (homogeneity of variances): Tollner 0.820; ΔTollner *p* = 0.892; SPS 0.212; ΔSPS *p* = 0.963; Log-CRP 0.450; ΔCRP *p* = 0.624

• Shapiro–Wilk normality of residuals: Tollner *p* = 0.225; SPS *p* = 0.159; Log-CRP *p* = 0.102

All models adjust for baseline (Day-0) values of the corresponding outcome

^a^ Pairwise comparisons are based on estimated marginal means with Bonferroni adjustment

^b^ CRP was analyzed using ANCOVA on log-transformed CRP at Day-7 adjusted for log-CRP at Day-0. Adjusted CRP values shown are geometric means obtained by back-transforming EMMs (exp). Comparisons are presented as geometric mean ratios (GMR) with 95% CIs from back-transformed log-scale differences

#### Modified Töllner score

For the day-7 Modified Töllner score, a significant group effect was observed (*F*(2,57) = 24.394;*p* < 0.001) with a large effect size (*ηp*^2^​=0.461). The adjusted mean for the control group was 9.35 (95% CI 7.94–10.75), which was significantly higher than the vitamin D 400 IU group (3.65; 95% CI 2.10–5.20) and the 800 IU group (2.72; 95% CI 1.17–4.27). Pairwise comparisons confirmed that both intervention doses produced significantly lower day-7 scores than the control (adjusted differences of 5.70 and 6.63, respectively; both *p* < 0.001), with no significant difference between the two vitamin D doses (*p* = 1.000). Analysis of the score change (Δ) remained consistent (*F*(2,58) = 23.964;*p* < 0.001;*ηp*^2^​=0.452); the control group showed an increase (Δ + 1.30), while the vitamin D groups showed significant reductions (Δ − 4.32 for 400 IU and − 5.47 for 800 IU; both against control).

#### Sepsis Prediction Score (SPS)

Analysis of the day-7 SPS revealed a highly significant group effect (*F*(2,57) = 25.738;*p* < 0.001,*ηp*^2^​=0.475). The adjusted mean for the control group was 3.62 (95% CI 3.07–4.18), compared to 1.01 (95% CI 0.41–1.62) in the 400 IU group and 1.08 (95% CI 0.48–1.67) in the 800 IU group. Both vitamin D groups differed significantly from the control (*p* < 0.001 for both), but were similar to each other (*p* = 1.000). The analysis of change (ΔSPS) further supported these findings (*F*(2,58) = 16.447;*p* < 0.001;*ηp*^2​^=0.362), where vitamin D groups showed a reduction of approximately 2 points (Δ − 2.05 and − 2.11) compared to a negligible change in the control group (Δ + 0.09; *p* < 0.001).

#### C-Reactive Protein (CRP)

For day-7 CRP levels (analyzed via ANCOVA on log-transformed data), no significant group effect was found (*F*(2,57) = 0.877;*p* = 0.421;*ηp*^2^​=0.030). Although geometric mean CRP levels appeared numerically lower in the 400 IU (0.56 mg/dL) and 800 IU (0.78 mg/dL) groups compared to control (1.29 mg/dL), pairwise comparisons yielded no statistical significance (Geometric Mean Ratio 0.43, and 0.60, *p* = 1.000, respectively). Similarly, the analysis of ΔCRP showed a trend toward reduction in the vitamin D groups (Δ − 0.67 and − 1.82) versus an increase in the control group (Δ + 2.56), but this did not reach statistical significance (*F*(2,58) = 2.618;*p* = 0.082;*ηp*^2^​=0.083).

#### Statistical validation

Model diagnostics confirmed the validity of the ANCOVA results. Homogeneity of variance was maintained across all models (Levene’s test: Töllner *p* = 0.820; SPS *p* = 0.212; Log-CRP *p* = 0.450), and the residuals for the day-7 outcomes followed a normal distribution (Shapiro–Wilk test: Töllner *p* = 0.225; SPS *p* = 0.159; Log-CRP *p* = 0.102).

### Adverse events monitoring

Adverse events were monitored daily throughout the 7-day intervention period. In the 800 IU group, reported events included vomiting (*n* = 3; 15.8%), abdominal distension (*n* = 1; 5.3%), and mild hypercalcemia (*n* = 5; 26.3%). These events were classified as Grade 1 (mild) and did not require cessation of the study protocol. The gastrointestinal symptoms were attributed to the scheduled increase in enteral feeding volumes rather than the study intervention. Similarly, the incidence of hypercalcemia was confounded by the concurrent administration of intravenous calcium as part of parenteral nutrition. In the 400 IU group, only vomiting was reported (*n* = 2; 10.5%), with no incidence of distension nor hypercalcemia (Table 4).

**Table 4:** Outcomes and adverse events of analyzed participants

Variable	Control (*n* = 23)	Vitamin D 400 IU (*n* = 19)	Vitamin D 800 IU (*n* = 19)	*p* value
Outcome, n (%)				< 0.001^a^*
Death	9 (39.1%)	1 (5.3%)	0 (0.0%)	
Discharge	14 (60.9%)	18 (94.7%)	19 (100.0%)	
Adverse events (valid *N* = 38; Vitamin D groups only)				
Vomiting, n (%)		2 (10.5%)	3 (15.8%)	1.000^b^
Abdominal distension (girth ↑ >2 cm), n (%)		0 (0.0%)	1 (5.3%)	1.000^b^
Hypercalcemia, n (%)		0 (0.0%)	5 (26.3%)	0.046 ^b^*

Numerical data p-values were calculated using.

^a^ Chi-square test

^b^ Fisher’s exact test

*Significant p-value < 0.05

### Clinical outcomes

All-cause mortality was significantly higher in the control group compared to the intervention groups (*p* < 0.001). In the control group, a total of 17 deaths occurred (17/35; 48.6%). Of these, 8 neonates died during the active 7-day study period and were excluded from the per-protocol analysis of sepsis scores and biomarkers due to missing post-baseline data. Among the 23 neonates in the control group who completed the 7-day intervention, the mortality rate was 39.1% (*n* = 9), with an average time of death occurring 13 days after the study period ended. In contrast, the 400 IU group reported 1 death (1/19; 5.3%) which occurred 10 days after the study period, and the 800 IU group reported 1 early death during the study period (1/23; 4.3%) with no subsequent mortalities. The remaining neonates were discharged with improvement (Table 4). There was no significant association between vitamin D levels and survival among preterm infants with sepsis (Table 5).

**Table 5:** Vitamin D levels and outcomes in preterm infants with sepsis

Vitamin D	Deceased (*n* = 8)	Alive (*n* = 15)	*p* value
25(OH)D, Median (IQR)	8.51 (5.43–28.26)	9.36 (3.61–13.13)	0.651^b^
Classification, n (%)			0.269^c^
Sufficient (*n* = 3)	2 (25.0%)	1 (6.7%)	
Insufficient (*n* = 0)	-	-	
Deficient (*n* = 20)	6 (75.0%)	14 (93.3%)	

Numerical data p-values were calculated using

^b^Mann-Whitney test

Categorical data p-values were calculated based on

^c^Chi-Square test

Significant p-value < 0.05

## Discussion

This randomized controlled trial demonstrated that adjuvant vitamin D administration, at both 400 IU and 800 IU daily, significantly improved clinical sepsis scores in preterm infants. While the groups were well-balanced regarding demographics and respiratory support, a notable disparity in baseline clinical severity was observed. Specifically, the control group exhibited a higher requirement for inotropic support and consisted exclusively of neonates kept NPO due to medical necessity. While these factors likely contributed to the higher mortality observed in the control arm, the significant improvement in the modified Töllner and SPS in the intervention groups provides a robust clinical signal that vitamin D may play a therapeutic role in neonatal sepsis management.

The microbiological landscape of this study was dominated by Gram-negative organisms, specifically *Acinetobacter baumanii* and *Klebsiella pneumoniae*, which is consistent with pathogen distributions in neonatal intensive care units within developing regions. The high prevalence of these multi-drug resistant pathogens underscores the severity of the sepsis cases managed in this trial. Importantly, antibiotic regimens were standardized across all groups, ensuring that the observed clinical improvements were attributable to the adjuvant intervention rather than variations in primary antimicrobial therapy. This consistency allows for a more focused interpretation of vitamin D’s role as an immunomodulatory adjuvant in the presence of standard-of-care antibiotics.

A high prevalence of vitamin D deficiency was observed, with 81.9% of participants presenting with low status (72.1% deficient; 9.8% insufficient). This rate reached 87% in the control group, who were strictly NPO at enrollment. These findings are even higher than the 61% prevalence reported in a comprehensive 2021 meta-analysis, yet they align with observations that vitamin D deficiency is significantly more common in sepsis-affected neonates (79.4%) compared to sepsis-free controls (43.7%) [10]. The high mortality and high deficiency rates observed in our control group reinforce the proposed relationship between vitamin D status and sepsis severity recently highlighted by Dua et al. [14]

Regarding clinical outcomes, vitamin D administration was associated with a marked decrease in sepsis scores, whereas scores in the control group remained stable or worsened. Notably, no significant difference was found between the 400 IU and 800 IU dose groups in terms of sepsis scores. Regarding CRP, it is important to highlight a nuanced finding: while the overall change (Δ) in CRP differed significantly between therapy groups, indicating a reduction in the inflammatory burden, the absolute ANCOVA-adjusted day-7 values did not demonstrate a statistically significant group effect (*p* = 0.421). This finding aligns closely with a similar randomized trial in Egypt, which also reported no significant difference between 400 IU and 800 IU doses regarding clinical outcomes or reductions in pro-inflammatory cytokines (IL-6 and TNF-α) [15]. While research by Toaima et al. in Nepal demonstrated faster recovery times in infants receiving 800 IU compared to controls [16], our results suggest that the standard 400 IU dose is equally efficacious in modulating the acute inflammatory response in preterm infants.

The equivalent efficacy of both doses suggests that massive systemic increases in serum 25(OH)D may not be required to achieve clinical benefit during acute illness. Although post-intervention vitamin D levels were not measured—as it is not yet fully established whether a 400 IU or 800 IU daily dose is sufficient to achieve a significant rise in serum 25(OH)D concentrations within a restricted 7-day intervention window—the rapid clinical response likely involves the immediate induction of antimicrobial peptides like cathelicidins and defensins. These molecules modulate the innate immune response before systemic homeostasis is fully achieved [17], and our data implies that a 400 IU dose is sufficient to trigger this beneficial local response, although it was previously known that an 800 IU dose better improved vitamin D status in preterm infants [18]. 

Furthermore, the immunomodulatory benefit of vitamin D extends to the adaptive immune system. It acts by inhibiting the differentiation of T-helper (Th)-1 and Th-17 cells, thereby reducing the production of pro-inflammatory cytokines such as IL-2, INF-γ, and TNF-α. Simultaneously, it promotes an anti-inflammatory state by increasing the production of IL-4, IL-10, and IL-13 through Th-2 and regulatory T-cell pathways [17, 19, 20]. When deficiency occurs, an imbalance arises, leading to an overproduction of pro-inflammatory cytokines that can exacerbate systemic inflammatory response syndrome (SIRS). The stabilization of sepsis scores and CRP in our intervention groups suggests that vitamin D helps restore this critical cytokine balance regardless of whether a 400 IU or 800 IU dose is utilized.

The selection of CRP as our primary inflammatory marker, rather than Procalcitonin (PCT), was a pragmatic decision based on the widespread availability and lower cost of CRP in our setting. While PCT is widely recognized as a superior biomarker for neonatal sepsis due to its higher sensitivity and more rapid kinetics, CRP remains the most feasible option in many clinical environments. The significant reduction in CRP observed in the 800 IU group provides measurable evidence of a reduced inflammatory burden. We acknowledge that the 7-day assessment window—necessitated by institutional sampling constraints—may not have captured the immediate kinetics of the inflammatory response, but it nonetheless established a clear clinical endpoint for the intervention.

The higher mortality observed in the control group (39.1%) must be interpreted with caution due to the baseline imbalance in cardiovascular stability. The control group’s higher inotrope requirement (69.6%) and NPO status suggest a more severe degree of initial circulatory failure. From an ethical standpoint, the control group comprised only NPO neonates because it was deemed unethical to withhold oral vitamin D from neonates who were already tolerating enteral nutrition. While this baseline acuity is a potential confounder for survival, it does not diminish the significant clinical improvement seen in the survivors’ sepsis scores, which remained statistically superior in the vitamin D arms regardless of initial severity.

Finally, while both doses were equally efficacious, safety monitoring revealed incidences of hypercalcemia exclusively in the 800 IU group (26.3%). Although all affected neonates were receiving concurrent intravenous calcium, which likely confounded this result, the lack of added clinical benefit from the 800 IU dose suggests that 400 IU may be the safer, preferred adjuvant dosage. Gastrointestinal symptoms, such as vomiting and distension, were transient and resolved with the management of enteral feeding volumes, suggesting they were not directly attributable to vitamin D toxicity. Future investigations should include long-term monitoring to further establish the optimal and safest dosing profile for adjuvant vitamin D in this vulnerable population.

### Limitations

This study has several limitations. First, the difference in nutritional status between groups presents a potential confounder. The control group consisted exclusively of neonates kept NPO due to medical necessity, while the intervention groups received enteral nutrition. This allocation was an ethical necessity, as it would be inappropriate to withhold standard-of-care vitamin D administration from neonates capable of enteral intake. While this reflects real-world clinical and ethical constraints, it complicates the isolation of vitamin D’s effects from the baseline acuity associated with NPO status.

Second, the selection of biomarkers and the timing of assessments were influenced by practical constraints. Although procalcitonin is recognized as a superior biomarker for neonatal sepsis due to its sensitivity and rapid kinetics, CRP was selected due to its widespread availability and lower cost in a resource-limited setting. Additionally, the 7-day interval for repeated CRP assessment—necessitated by institutional blood sampling limitations and laboratory resource constraints—may not have captured the immediate kinetics of the inflammatory response.

Third, while the sample size was sufficient to detect primary outcomes, randomization inadvertently resulted in a baseline imbalance in requirement for inotropic support. Although ANCOVA was utilized to adjust for baseline sepsis scores in our primary outcome analysis, this underlying discrepancy restricts our ability to draw definitive conclusions regarding survival outcomes.

Finally, this trial was retrospectively registered on ClinicalTrials.gov due to administrative constraints and the need to expedite data collection within a strictly time-bound academic research program. Importantly, full institutional ethical clearance was secured prior to the enrollment of the first subject. Future larger-scale, multicenter randomized controlled trials are required to ensure a more even distribution of baseline severity and further validate these findings.

## Conclusions

In preterm infants with sepsis, vitamin D administration was associated with significant improvements in the modified Töllner sepsis score and Sepsis Prediction Score, with comparable effectiveness between the 400 IU and 800 IU doses. These findings support the potential role of vitamin D as an adjuvant therapy in preterm infants with neonatal sepsis, but require further confirmation in larger, more robust studies.

**Figure:**
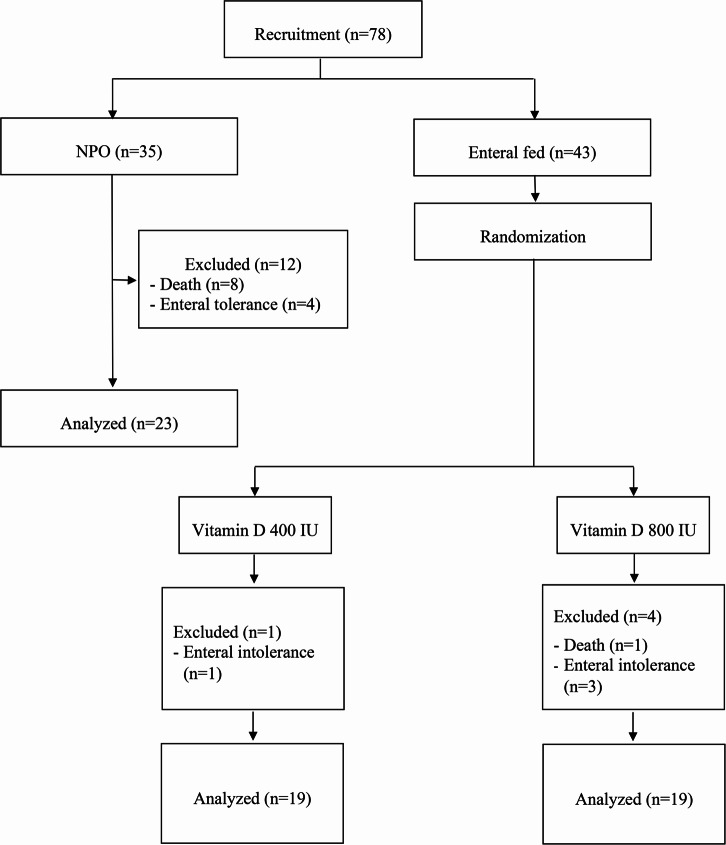
Flow diagram

## Supplementary Information

Supplementary Material 1.

## Data Availability

The raw data supporting the conclusions of this article will be made available by the authors, without undue reservation. The full trial protocol is available in the trial registry at ClinicalTrials.gov under identifier NCT07245277.

## Abbreviations

ANCOVA: Analysis of Covariance
CI: Confidence Interval
CONSORT: Consolidated Standards of Reporting Trials
CRP: C-reactive protein
ELISA: Enzyme-Linked Immunosorbent Assay
IFN: Interferon
IL: Interleukin
IVH: Intraventricular Hemorrhage
MRCoNS: Methicillin-Resistant Coagulase-Negative Staphylococci
NEC: Necrotizing Enterocolitis
NHCU: Neonatal High Care Unit
NICU: Neonatal Intensive Care Unit
NPO: nil per os
PCT: Procalcitonin
RDS: Respiratory Distress Syndrome
SIRS: Systemic Inflammatory Response Syndrome
SPS: Sepsis Prediction Score
Th: T-helper
TNF: Tumor Necrosis Factor
UNIANOVA: Univariate Analysis of Variance

## References

[1] GBD 2017 Collaborators. Global, regional, and national incidence, prevalence, and years lived with disability for 354 diseases and injuries for 195 countries and territories, 1990–2017: a systematic analysis for the Global Burden of Disease Study 2017. Lancet. 2018;392(10159):1789–858. 30496104 10.1016/S0140-6736(18)32279-7 PMC6227754

[2] Kamsiah K, Hasibuan BS, Arto KS. The relationship between vitamin D levels and clinical outcomes of neonatal sepsis in Haji Adam Malik Hospital Medan, Indonesia. Open Access Maced J Med Sci. 2021;9(B):698–703.

[3] Al-Wassia HK, Saeedi FA. Incidence and risk factors of early-onset sepsis among preterm infants in a teaching hospital: a retrospective cohort study. J Clin Neonatol. 2024;13(4):131–6.

[4] Raymond SL, Rincon JC, Wynn JL, Moldawer LL, Larson SD. Impact of early-life exposures to infections, antibiotics, and vaccines on perinatal and long-term health and disease. Front immunol. 2017;8:729. 10.3389/fimmu.2017.00729. 28690615 10.3389/fimmu.2017.00729 PMC5481313

[5] Turgut M, Özdemir ÖMa, Erdal B. Evaluation of EMA, Töllner and Rodwell scores in the diagnosis of neonatal sepsis. Pam Med J. 2024;17(4):746–54.

[6] Harsanti A, Sekarwana N, Rusmil K. Perbedaan skor sepsis modifikasi Töllner dan kadar prokalsitonin serum sebelum dengan setelah pemberian antibiotik empiris pada sepsis neonatorum. Sari Pediatri. 2014;16(3):178–8218.

[7] Schüller SS, Kramer BW, Villamor E, Spittler A, Berger A, Levy O. Immunomodulation to prevent or treat neonatal sepsis: past, present, and future. Front pediatr. 2018;6. 10.3389/fped.2018.00199. 10.3389/fped.2018.00199 PMC6060673 30073156

[8] Henderson R, Kim S, Lee E. Use of melatonin as adjunctive therapy in neonatal sepsis: A systematic review and meta-analysis. Complement Ther Med. 2018;39:131–6. 30012383 10.1016/j.ctim.2018.06.002

[9] Abiramalatha T, Ramaswamy VV, Bandyopadhyay T, Somanath SH, Shaik NB, Kallem VR, et al. Adjuvant therapy in neonatal sepsis to prevent mortality - A systematic review and network meta-analysis. J Neonatal Perinat Med. 2022;15(4):699–719. 10.3233/NPM-221025 36189501

[10] Workneh BZ, Worku T, Alemu A. Effects of vitamin D on neonatal sepsis: A systematic review and meta-analysis. Food Sci Nutr. 2021;9(1):375–88. 33473300 10.1002/fsn3.2003 PMC7802542

[11] Oktaria V, Graham SM, Triasih R, Soenarto Y, Bines JE, Ponsonby AL, et al. The prevalence and determinants of vitamin D deficiency in Indonesian infants at birth and six months of age. PLoS ONE. 2020;15(10):e0239603. 10.1371/journal.pone.0239603. 33017838 10.1371/journal.pone.0239603 PMC7535980

[12] Seymen-Karabulut G, Günlemez A, Gökalp AS, Hatun Ş, Kaya Narter F, Mutlu M, et al. Vitamin D Deficiency Prevalence in Late Neonatal Hypocalcemia: A Multicenter Study. J Clin Res Pediatr Endocrinol. 2021;13(4):384–90. 34013710 10.4274/jcrpe.galenos.2020.2021.0169 PMC8638626

[13] Hagag AA, El Frargy MS, Houdeeb HA. Therapeutic Value of Vitamin D as an Adjuvant Therapy in Neonates with Sepsis. Infect Disord Drug Targets. 2020;20(4):440–7. 31241441 10.2174/1871526519666190626141859

[14] Dua J, Jadhav RS, Bahal M, Mane S, Kale S, Garlapati S, et al. Association between vitamin d deficiency and sepsis in term neonates: a case-control study. Cureus. 2024;16(10):e72468. 10.7759/cureus.72468. 39600767 10.7759/cureus.72468 PMC11590038

[15] Abdel-Hady H, Yahia S, Megahed A, Mesbah A, Seif B, Nageh E, et al. Vitamin D and inflammatory mediators in preterm infants with late-onset sepsis: a randomized controlled trial. J Pediatr Gastroenterol Nutr. 2019. 10.1097/MPG.0000000000002238. 30896608 10.1097/MPG.0000000000002238

[16] Toaima N, Ali M, Abdelsattar H, Ahmad M, Abdelaal N. Role of vitamin D therapy in recovery from early onset neonatal sepsis – a randomized controlled trial. J Nepal Paediatr Soc. 2024;44:36–42.

[17] Chirumbolo S, Bjørklund G, Sboarina A, Vella A. The role of vitamin D in the immune system as a pro-survival molecule. Clin Ther. 2017;39(5):894–916. 28438353 10.1016/j.clinthera.2017.03.021

[18] Rueang-Amnat N, Kittisakmontri K, Khuwuthyakorn V, Kosarat S, Manopunya S, Pomrop M. Improving vitamin d status in preterm newborns: a randomized trial of 800 vs. 400 IU/day. Nutrients. 2025;17(11). 10.3390/nu17111888. 10.3390/nu17111888 PMC12158240 40507157

[19] Sassi F, Tamone C, D’Amelio P. Vitamin D: Nutrient, hormone, and immunomodulator. Nutrients. 2018;10(11). 10.3390/nu10111656. 10.3390/nu10111656 PMC6266123 30400332

[20] Gamal TS, Madiha AS, Hanan MK, Abdel-Azeem ME, Marian GS. Neonatal and maternal 25-oh vitamin d serum levels in neonates with early-onset sepsis. Child (Basel). 2017;4(5). 10.3390/children4050037. 10.3390/children4050037 PMC5447995 28486434

